# Comparison of Long-Term Survival of Patients with Solitary Large Hepatocellular Carcinoma of BCLC Stage A after Liver Resection or Transarterial Chemoembolization: A Propensity Score Analysis

**DOI:** 10.1371/journal.pone.0115834

**Published:** 2014-12-26

**Authors:** Shao-Liang Zhu, Yang Ke, Yu-Chong Peng, Liang Ma, Hang Li, Le-Qun Li, Jian-Hong Zhong

**Affiliations:** 1 Department of Hepatobiliary Surgery, Affiliated Tumor Hospital of Guangxi Medical University, Nanning 530021, China; 2 Department of Hepatobiliary Surgery, the Second Affiliated Hospital of Kunming Medical University, Kunming 650101, China; 3 Department of Ultrasound, Affiliated Tumor Hospital of Guangxi Medical University, Nanning 530021, China; Xiangya Hospital of Central South University, China

## Abstract

**Background:**

The aim of this study was to compare the long-term outcome of patients with a solitary large (>5 cm) hepatocellular carcinoma (HCC) in Barcelona Clinic Liver Cancer (BCLC) stage A who received liver resection (LR) or transarterial chemoembolization (TACE).

**Methods:**

Our study examined 128 patients treated by LR and 90 treated by TACE. To reduce bias in patient selection, we conducted propensity score analysis in the present study and 54 pairs of patients after propensity score matching were generated, their long-term survival was compared using the Kaplan–Meier method. Independent predictors of survival were identified by multivariate analysis.

**Results:**

Long-term survival was significantly better for the LR group by log-rank test (P<0.001). In multivariate analysis, tumor size, serum ALT level and TACE independently predicted survival. Despite similar baseline characteristics after propensity score matching, LR group still had significantly better survival (1 year, 68.5 vs. 55.0%; 3 years, 47.6 vs. 21.2%; 5 years, 41.3 vs. 18.5%; *P* = 0.007) than TACE group. The LR and TACE groups had comparable 30- and 90-day post-treatment mortality. Multivariate analysis showed that serum ALT level, serum AFP level and TACE independently predicted survival by multivariate analysis after propensity score matching.

**Conclusion:**

Our propensity-score-matched study suggested that LR provided significantly better long-term survival than TACE for a solitary large HCC of the BCLC stage A, regardless of tumor size.

## Introduction

Hepatocellular carcinoma (HCC) is the fifth most common malignancy worldwide and ranks as the second leading cause of cancer death in males and the sixth leading cause of cancer death in women worldwide [1]–[4]. Because of the high postoperative recurrence rate and high cancer mortality, the prognosis of HCC patients is discouraging. Thus, identifying the optimal therapy for HCC patients plays a significant role in maximizing their long-term survival.

Studies have validated and proposed the clinical usefulness of BCLC staging system [5]–[7], making it one of the most reliable for HCC. According to the system, patients who have Child-Pugh A liver function and early-stage HCC involving either a single tumor of any size or 2–3 tumors ≤3 cm belongs to BCLC stage A and Liver resection (LR) is defined as first-line treatment for this stage, especially for a solitary HCC [6]. Transarterial chemoembolization (TACE) is recommended for stage B HCC, where it is associated with better 2-year survival than conservative treatment, but it is not recommended for stage A HCC [6]. While some authors have reported TACE to be associated with good survival in patients with stage A HCC [8]–[10]. On the other hand, some authors have reported that LR provides better long-term survival than TACE for HCC patients of BCLC stage A [11], [12]. Therefore, management of HCC in BCLC stage A is still controversial and need more investigation. However, studies of this kind are quite limited, especially for patients with a solitary large (>5 cm) HCC in BCLC stage A.

Therefore in this study, we investigated the long-term survival of patients with HCC with a solitary large (>5 cm) HCC in BCLC stage A who received LR and TACE by conducting propensity score matching to select matched pairs of HCC patients. In addition, independent factors associated with prognosis were determined, and post-treatment complications and mortality were also analyzed.

## Patients and Methods

### Ethics Statement

First, this study was conducted in accordance with the Declaration of Helsinki. Secondly, written informed consent was given by all participants for their clinical records to be used in this study. Lastly, it was approved by the Institutional Review Board of Affiliated Tumor Hospital of Guangxi Medical University.

### Patients

This retrospective study examined data collected on patients with a solitary large HCC at our hospital between January 2003 and September 2007. Neither vascular invasion nor distant metastasis was observed in these patients, and all were classified as BCLC stage A with Child-Pugh class A or B. Patients who satisfied the indications for both LR and TACE were treated with LR unless the patients requested TACE. Patients who received their initial HCC treatment at other centers were excluded. We excluded patients who received only local ablation therapy ethanol injection or supportive care. HCC diagnosis was confirmed after LR by histopathological examination of surgical samples in all patients. HCC diagnosis was confirmed in TACE patients by needle biopsy or by analysis of two images [ultrasonography, computed tomography (CT), or magnetic resonance imaging] in conjunction with a serum level of a-fetoprotein (AFP) higher than 400 ng/mL. Needle biopsy was performed in patients whose diagnosis based on imaging and AFP level was uncertain.

### Propensity Score Matching

In order to reduce the bias in patient selection, propensity score analysis was developed to investigate causal relationships between treatments and outcomes in a retrospective study other than a randomized controlled trial [13]. Propensity score matching (PSM) was used to generate a matched pairs of patients to compare long-term survival between patients undergoing LR and TACE. Propensity scores were estimated using a logistic regression model based on age, gender, tumor size, hepatitis B virus (HBV) infection status, Child-Pugh class, cirrhosis, total bilirubin, serum AFP level, alanine aminotransferase (ALT), aspartate aminotransferase (AST), prothrombin time, albumin and platelet count. One-to-one matching without replacement was performed using a 0.1 caliper width, and the resulting score-matched pairs were used in subsequent analyses as indicated [14].

### Liver Resection

Indications for surgery were lack of ascites, hepatic encephalopathy, and hypersplenism, as well as the presence of appropriate residual liver volume, as determined by volumetric computed tomography [15]. The LR technique was performed as described. [11], [16] The clinicopathological data for these patients are summarized in Table 1.

**Table 1:** Comparison of preoperative clinicopathologic data of patients receiving liver resection (LR) or transarterial chemoembolization (TACE).

Variable	Liver resection (n = 128)	TACE (n = 90)	*P* value
Age (year)	45.4±12.7	48.7±11.8	0.503
Gender (M/F), n (%)	118 (92.2%)/10 (7.8%)	86 (95.6%)/4 (4.4%)	0.318
Tumor size (cm)	7.9±2.4	10.1±2.7	0.102
HbsAg (+), n (%)	123 (96.1%)	89 (98.9%)	0.411
Child-Pugh class, n (%)			
A	127 (99.2%)	87 (96.7%)	0.384
B	1 (0.8%)	3 (3.3%)	
Cirrhosis	117 (91.4%)	82 (92.2%)	0.939
AFP (ng/ml), n (%)			
≥400	53 (41.4%)	44 (48.9%)	0.274
<400	75 (58.6%)	46 (51.1%)	
Total bilirubin (µmol/L)	13.1±5.5	16.0±7.8	0.001
ALT (U/L)	53.8±86.6	67.7±47.5	0.628
AST (U/L)	42.8±22.2	51.1±28.9	0.192
Prothrombin time (s)	13.1±1.6	13.5±1.8	0.081
Albumin (g/L)	39.5±4.3	38.3±4.3	0.923
Platelet count (10^9^/L)	197.8±77.5	203.9±77.2	0.901
Esophageal varices, n (%)			
Presence	17 (13.3%)	19 (21.1%)	0.125
Absence	111 (86.7%)	71 (78.9%)	
Postoperative complications, n (%)	31 (24.2%)	14 (15.6%)	0.120
30-day mortality, n (%)	0 (0)	2 (2.2)	0.169
90-day mortality, n (%)	5 (3.9)	8 (8.9)	0.126

Values with “±” are written as mean ± SD.

AFP, alpha-fetoprotein; ALT alanine, aminotransferase; AST, aspartate aminotransferase.

### TACE Procedure

Although all the patients in this study have opportunities to receive surgery according to BCLC stage system, parts of them required TACE treatment, because of worrying about postoperative complications or other reasons. With the patient under local anesthesia, a 4F-to-5F French catheter was introduced into the abdominal aorta via the superficial femoral artery using the Seldinger technique. Hepatic arterial angiography was performed using fluoroscopy to guide the catheter into the celiac and superior mesenteric artery. Then the feeding arteries, tumor stain, and vascular anatomy surrounding the tumor were identified. A microcatheter was introduced through the 4F-to-5F catheter to the feeding arteries. An emulsion of 5–15 ml lipiodol (Andre Guerbet, Aulnay-sous-Bois, France) and 5-fluorouracil (500 mg/m^2^) with or without adriamycin (30 mg/m^2^) was infused into the feeding arteries. Thereafter, the feeding hepatic artery was embolized with Gelfoam, which is an absorbable cubic gelatin sponge particles. After the procedure, the doctor removes the catheter and sheath and applies pressure to the entry site for 5 to 20 minutes to prevent bleeding. The patient remains on bed rest overnight and is discharged the next day. If complications occur, the patient must be kept in hospital for several days to manage them [17]. A follow-up CT scan was arranged one month later to evaluate the effect of TACE. The course was repeated once every 1–2 months for 2–6 cycles.

### Follow-Up

After treatment, follow-up of all surviving patients included a liver function test, estimation of serum α-fetoprotein levels, dynamic liver computed tomography (CT), MRI, liver ultrasonography and chest radiography at an interval of 2–3 months, especially during the first 2 years. Overall survival was calculated from the day of surgery until the date of the last follow up.

### Statistical Analysis

Continuous variables are expressed as mean ± standard deviation and compared using the t test. Categorical variables were compared using the chi-square test or Fisher exact test, where appropriate. Survival analysis was calculated by the Kaplan–Meier method and group results were compared using the log-rank test. Multivariate analysis to identify independent prognostic factors was carried out using the Cox proportional hazards mode. All statistical analyses were performed with SPSS (version 19.0, Chicago, IL, USA). For all tests, a *P* value <0.05 was considered statistically significant.

## Result

### Clinicopathological Data of All Patients

Between January 2003 and September 2007, 815 consecutive patients were initially diagnosed as having a solitary large HCC at our hospital. Of these patients, 231 patients were excluded because they had received initial HCC treatment at other centers. Among the remaining 584 patients, 275 had BCLC stage A HCC. Of these, 57 were excluded because they received only local ablation therapy, ethanol injection or supportive care. Therefore, the clinical database of 218 patients was retrospectively analyzed. Of these patients, 128 patients were treated by LR and 90 were treated by TACE.

The comparison of clinicopatholgic characteristics between the LR group and TACE group is shown in table 1.Most clinical characteristics were similar between the two groups. There were no significant differences in age, tumor size, gender ratios, proportion of HBsAg positive, levels of AFP, albumin, ALT, AST, or prothrombin time. However, Patients in the TACE group had higher total serum bilirubin (*P* = 0.001) than those in the LR group. In addition, More than 90% of patients were male and HBsAg positive in both groups.

### Survival Analysis of All Patients

**Figure 1:**
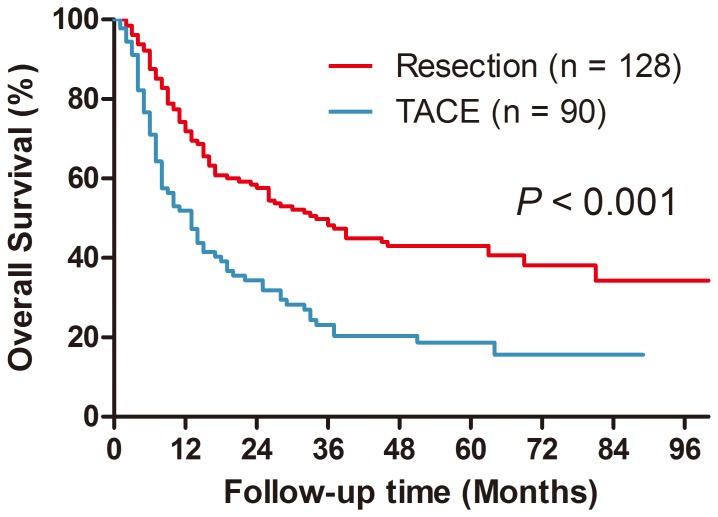
Overall survival curves for the liver resection (LR) group and the transarterial chemoembolization (TACE) group.

The comparison of long-term survival between patients undergoing LR and TACE is shown in Fig. 1.Median follow-up was 34.0 months in the LR group and 19.0 months in the TACE group. During follow-up, 64 (50.2%) patients in the LR group and 47 (52.7%) patients in the TACE group died, respectively. The 1-, 3-, and 5-year survival rates of patients receiving LR and TACE were 71.9% vs. 51.9%, 48.2% vs. 23.1%, and 43.0% vs. 18.7%, respectively (*P*<0.001). Univariate analysis identified the following prognostic factors that predicted increased risk of mortality: tumor size, serum AFP level ≥400 ng/ml, serum ALT level, serum albumin level and TACE treatment. Multivariate analysis showed that tumor size (HR = 1.066, 95% CI: 1.002–1.134, *P* = 0.043), serum ALT level (HR = 1.002, 95% CI: 1.000–1,004, *P* = 0.042), and TACE treatment (HR = 1.637, 95% CI: 1.148–2.334, *P* = 0.006) were independent prognostic factors.

### Clinicopathological Data of Propensity-Score-Matched Patients

Patients treated by LR or TACE were matched one-to-one using PSM for minimizing the confounding factors. Clinical variables entered after PSM were age, gender, tumor size, hepatitis B virus (HBV) infection status, Child-Pugh class, cirrhosis, total bilirubin, serum AFP level, alanine aminotransferase (ALT), aspartate aminotransferase (AST), prothrombin time, albumin and platelet count. Fifty-four pair patients were matched in each group. The above thirteen factors appeared to be well matched showed no significant differences between groups (Table 2).

**Table 2:** Comparison of preoperative clinicopathologic data of patients receiving liver resection (LR) or transarterial chemoembolization (TACE) after propensity score matching (PSM).

Variable after PSM	Liver resection (n = 54)	TACE (n = 54)	*P* value
Age (year)	46.7±13.8	48.1±12.3	0.592
Gender (M/F), n (%)	49 (90.7%)/5 (9.3%)	50 (92.6%)/4 (7.4%)	1.000
Tumor size (cm)	8.8±2.7	9.0±2.0	0.672
HbsAg (+), n (%)	50 (92.6%)	53 (98.1%)	0.360
Child-Pugh class, n (%)			
A	54 (100%)	54 (100%)	1.000
B	0 (0%)	0 (3%)	
Cirrhosis	52 (94.5%)	53 (96.4%)	1.000
AFP (ng/ml), n (%)			
≥400	22 (40.7%)	25 (46.3%)	0.507
<400	33 (59.3%)	29 (53.7%)	
Total bilirubin (µmol/L)	14.3±5.8	14.2±6.7	0.993
ALT (U/L)	72.1±134.9	66.2±45.0	0.761
AST (U/L)	48.4±26.9	49.5±30.5	0.833
Prothrombin time (s)	13.5±2.0	13.4±1.8	0.906
Albumin (g/L)	38.7±4.0	39.3±3.3	0.399
Platelet count(10^9^/L)	207.9±75.0	206.4±77.0	0.916
Esophageal varices, n (%)			
Presence	7 (13.0%)	8 (14.8%)	0.781
Absence	47 (87.0%)	46 (85.2%)	
Postoperative complications, n (%)	11 (20.4%)	9 (16.7%)	0.620
30-day mortality, n (%)	0 (0%)	1 (1.9%)	1.000
90-day mortality, n (%)	3 (5.6%)	3 (5.6%)	1.000

Values with “±” are written as mean ± SD.

PSM, propensity score matching; AFP, alpha-fetoprotein; ALT, alanine aminotransferase; AST, aspartate aminotransferase.

### Overall Survival Analysis of Propensity-Score-Matched Patients

**Figure 2:**
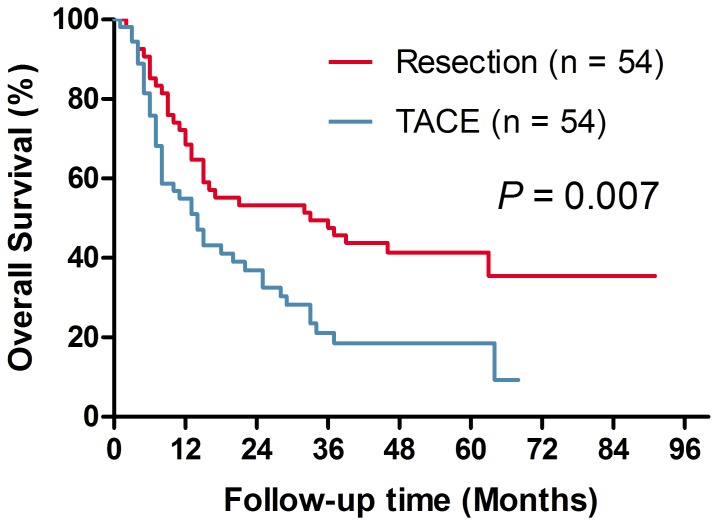
Overall survival curves for the liver resection (LR) group and the transarterial chemoembolization (TACE) group after propensity score matching.

Comparison of long-term survival between the two groups after PSM is shown in Fig. 2. After matching, the overall survival rates of the LR group were also better than that of the TACE group, with 68.5% vs. 55.0%, 47.6% vs. 21.2%, and 41.3% vs. 18.5%, respectively, for the 1-, 3- and 5-year overall survival rates (*P* = 0.007). Univariate analysis identified the following prognostic factors that predicted increased risk of mortality: serum AFP level ≥400 ng/ml, serum ALT level and TACE treatment (Table 3). In multivariate analysis, serum AFP level ≥400 ng/ml (HR = 1.870, 95% CI: 1.173–2.980, *P* = 0.009), serum ALT level (HR = 1.003, 95% CI: 1.000–1.005, *P* = 0.022) and TACE treatment (HR = 1.955, 95% CI: 1.221–3.130, *P* = 0.005) were still identified as independent predictors of poor prognosis (Table 3).

**Table 3:** Univariate and multivariate analysis to identify factors that predict overall survival in all patients and patients after propensity score matching (PSM).

Variables	All patients (n = 218)			Patients after PSM (n = 108)		
	HR	95% CI	*P* value	HR	95% CI	*P* value
Univariate analysis						
Age (year)	0.992	0.979–1.006	0.270	0.981	0.962–1.001	0.057
Gender (M/F)	1.311	0.613–2.801	0.485	1.013	0.408–2.519	0.977
Tumor size (cm)	1.108	1.050–1.169	<0.001	1.006	0.918–1.103	0.749
HbsAg (+/−)	1.500	0.478–4.709	0.488	1.220	0.384–3.879	0.736
Cirrhosis (presence/absence)	1.227	0.828–1.817	0.307	1.136	0.634–2.037	0.668
AFP (ng/mL) (≥400/<400)	1.410	1.018–1.954	0.039	1.681	1.064–2.665	0.026
Total bilirubin (µmol/L)	1.024	0.999–1.050	0.056	1.004	0.969–1.040	0.833
ALT (U/L)	1.002	1.000–1.004	0.011	1.002	1.000–1.004	0.044
AST (U/L)	1.001	0.994–1.007	0.811	1.002	0.994–1.009	0.689
Prothrombin time (s)	1.026	0.652–1.318	0.566	0.995	0.884–1.119	0.928
Albumin (g/L)	0.962	0.927–0.998	0.038	0.994	0.934–1.058	0.846
Platelet count (10^9^/L)	1.001	0.999–1.003	0.343	1.002	0.999–1.005	0.201
Treatment modality (Surgery/TACE)	2.006	1.445–2.784	<0.001	1.855	1.164–2.956	0.009
Multivariate analysis						
Tumor size (cm)	1.066	1.002–1.134	0.043			
AFP (ng/mL) (≥400/<400)	1.400	1.001–1.958	0.051	1.870	1.173–2.980	0.009
ALT (U/L)	1.002	1.000–1.004	0.042	1.003	1.000–1.005	0.022
Albumin (g/L)	0.974	0.937–1.013	0.189			
Treatment modality (Surgery/TACE)	1.637	1.148–2.334	0.006	1.955	1.221–3.130	0.005

PSM, propensity score matching; AFP, alpha-fetoprotein; ALT, alanine aminotransferase; AST, aspartate aminotransferase; HR, hazard ratio; 95% CI, 95% confidence interval.

### Mortality and Morbidity

Two patients in the TACE group died within 30 days of treatment. None of the patients underwent LR died in the same period. The 90-day mortality rate in the LR group (5 of 128, 3.9%) was similar with that in the TACE group (8 of 90, 8.9%). The incidence of postoperative complications was similar between these two groups (*P* = 0.120). And the most common complication of LR was pulmonary infection (5.5%), while liver failure (3.3%) was the major complication of TACE.

After PSM, only one patient died who underwent TACE died within 30 days of treatment, and the 90-day mortality rate had no difference between the LR group (3 of 54, 5.6%) and the TACE group (3 of 54, 5.6%; *P* = 1.000). The incidence of postoperative complications was also similar between these two groups (*P* = 0.620) after PSM. The specific complications of the two group patients are listed in Table 4.

**Table 4:** Postoperative complications of all patients and patients after propensity score matching (PSM).

Complication, n (%)	All patients		Patients after PSM	
	Liver resection (n = 128)	TACE (n = 90)	Liver resection (n = 54)	TACE (n = 54)
Pulmonary infection	7 (5.5)	3 (3.3)	5 (9.3)	2 (3.7)
Wound infection	2 (1.6)	0 (0)	1 (1.9)	0 (0)
Bile fistula	2 (1.6)	0 (0)	0 (0)	0 (0)
Abdomen infection	4 (3.1)	1 (1.1)	1 (1.9)	0 (0)
Liver failure	1 (0.8)	5 (5.6)	0 (0)	4 (7.4)
Bleeding	4 (3.1)	0 (0)	0 (0)	0 (0)
Gastrointestinal hemorrhage	2 (1.6)	1 (1.1)	0 (0)	1(1.9)
Intestinal obstruction	2 (01.6)	0 (0)	1 (1.9)	0 (0)
Pulmonary embolism	1 (0.8)	0 (0)	0 (0)	0 (0)
Incision dehiscence	3 (2.3)	0 (0)	2 (3.7)	0 (0)
Liver abscess	1 (0.8)	0 (0)	1 (1.9)	0 (0)
Puncture hematoma	0 (0)	4 (4.4)	0 (0)	2 (3.7%)
Peptic ulcer bleeding	2 (1.6)	0 (0)	0 (0)	0 (0)
Total	31 (24.2)	14 (15.6)	11 (20.4)	9 (16.7)

PSM, propensity score matching.

## Discussion

Since the introduction of the sixth AJCC tumor staging system a single large HCC without vascular invasion is no longer considered an unfavorable tumor [18], [19]. Moreover, the BCLC staging system which is widely used defines solitary HCC as early stage disease regardless of tumor size and the current practice guidelines recommend LR for solitary HCC without vascular invasion in patients with Child-Pugh class A or B [6], [7], [20]. However, these guidelines do not set a maximum size for solitary tumors. Recent studies have reported that LR provides better long-term survival than TACE for HCC patients beyond the Milan criteria (>5 cm) [21] and that solitary large HCC (>5 cm) was defined as a specific subtype, but exhibited a similar long-term outcome with small HCC after LR [22], [23]. While some authors reported that LR as the treatment for patients with large HCC provided significant poorer long-term survival than small HCC, moreover, accompanied by more incidence of surgical blood loss, subsequent liver decompensation and tumor recurrence [24]–[26]. Thus, the effectiveness of surgery compared with TACE for solitary large HCC remains controversial and need more investigation. Furthermore, these studies above involved various potential confounding factors on post-treatment outcomes, such as such as Child–Pugh class C, vascular invasion, multiple tumors, or BCLC stage B, C, or D [11], [21]–[26]. And more similar studies of surgery versus TACE in the context of long-term survival benefit for patients with a solitary large HCC in BCLC stage A are required for further investigation.

In this study, we compared post-treatment overall survival for LR or TACE in patients with a solitary large (>5 cm) HCC in BCLC stage A. These patients had Child-Pugh A or B liver function, without evidence of vascular invasion or extrahepatic spread. We found LR was associated with significantly higher survival rates, not only across all patients but also across patients after PSM. The results of survival benefit of LR for a solitary HCC in BCLC stage A are in accordance with that of Jin Y J et al. [12], whose study suggested that LR provides significantly longer survival than TACE for patients with a solitary HCC in BCLC stage A.

LR is traditionally associated with 5-year survival rate >50% in patients with early-stage HCC who meet the Milan criteria, and the BCLC staging system recommends LR as a first-line treatment [27], [28]. Jin Y J et al. reported that solitary large HCC (>5 cm) in BCLC stage A, despite beyond the Milan criteria (>5 cm), could have a 45.0% survival rate after LR which was significant higher than that after TACE (17.5%) [12]. Similarly, in the present study, we achieved a long-term survival rate of 41.3% after LR which was significant higher than that after TACE (18.5%) after PSM. Those observations support current guidelines for HCC treatment of solitary tumors regardless of tumor size in early stage patients according to the BCLC staging system and indicate that LR should be the first-line therapy for patients with a solitary large HCC in BCLC stage A. Liver transplantation is known to be the best treatment for HCC patients who meet the Milan criteria, with a 5-year survival rate >70% [29]–[32]. The enrolled patients in the present study with a single HCC >5 cm were not candidates for liver transplantation according to the Milan criteria. However recurrence of HCC after LR would be a candidate for liver transplantation if the intrahepatic recurrence were within the Milan criteria and patients can get the chance to receive liver transplantation if TACE can reduce tumor size [12].

Traditionally, LR for HCC was provided only to patients with smaller tumor size. LR for large tumor is technically difficult and usually requires major hepatic resection, which may be associated with the high risk of high mortality and poor prognosis. However, the surgical technique has been refined gradually in recent years. The surgical mortality rate reported by previous studies in patients with large HCC who received LR ranged from 0 to 6.9% [33]. In the present study, it can be observed that patients undergoing LR or TACE after PSM had similar 30-day (0% vs. 1.9%) and 90-day (5.6% vs. 5.6%) mortality, and both treatments offer acceptable and similar morbidity (20.4% vs. 16.7%) after PSM. The most common complication of LR was not liver function failure but unexpected pulmonary infection after PSM. While liver function failure was the major complication of TACE after PSM. Those observations suggested that LR can be considered a safe approach for the treatment of a solitary large HCC.

In order to identify as many prognostic factors as possible, we performed survival analysis by taking into account numerous factors previously shown to correlate with overall survival of HCC patients. Multivariate analysis identified serum ALT level, serum AFP level ≥400 ng/ml and TACE as independent prognostic factors after PSM. AFP is a biomarker widely used to diagnose HCC [34] and has even proven useful as a marker for predicting intrahepatic recurrence and extrahepatic metastasis [35], [36] and evaluating antitumor response after radiofrequency ablation [37] and sorafenib therapy [38]. Besides, several studies have suggested serum AFP level is an independent predictor of mortality in BCLC stage A HCC [11], [39]. Furthermore, several European and Japanese reports have stressed the importance of preoperative AFP levels by incorporating the AFP level into clinical prognostic scores [40], [41]. Jin Y J et al. found a larger tumor size (≥8 cm) compared with a smaller (5–8 cm) size was a significant predictor of post-treatment mortality in their study [12]. However, tumor size was not a prognostic factor after PSM in the present study. Therefore, larger sample and more investigation are required to explore whether there is a newly applicable maximum size in HCC of BCLC stage A that is a candidate for LR. And notably, TACE was confirmed a significant predictor associated with poor prognosis compared to LR after adjusting for other confounding factors.

The current study has some limitations. Firstly, it was a single-center study performed in the Asia-Pacific region with significantly higher prevalence of hepatitis B virus infection (>90%) than most western countries. Therefore external validation is needed from other study groups. Secondly, the inclusion period was between 2003 and 2007. We used 5-fluorouracil as the chemotherapeutic agent, which was uncommon used nowadays. Therefore, if new chemotherapeutic agents were used, patients may achieve better survival. Thirdly, the enrolled HCC patients with a marginally reserved hepatic function need to be carefully selected for LR because of the risk of treatment-related morbidity. Several patients (4/218, 1.8%) with a Child-Pugh class B HCC were enrolled in the present study. However, none of patients were enrolled after PSM. Lastly, the retrospective nature made this study vulnerable to potential bias, even after PSM, these biases still may not be completely avoided. Therefore in the future, we would expand our sample and prospective, randomized control trials would be performed for further research to revalidate the results of our study.

In conclusion, our propensity-score-matched findings indicated that LR may offer better long-term survival than TACE in patients with a solitary large (>5 cm) HCC of the BCLC stage A, regardless of tumor size. Therefore, HR should be considered as a first-line therapy for these patients. Prospective, randomized, control trials with large sample size are required to confirm the findings.
